# Targeting of PI3K/AKT signaling and DNA damage response in acute myeloid leukemia: a novel therapeutic strategy to boost chemotherapy response and overcome resistance

**DOI:** 10.20517/cdr.2021.76

**Published:** 2021-11-10

**Authors:** Montserrat Estruch, Camilla Vittori, Teresa Muñoz Montesinos, Kristian Reckzeh, Kim Theilgaard-Mönch

**Affiliations:** ^1^The Finsen Laboratory, Rigshospitalet/National University Hospital, Faculty of Health and Medical Sciences, University of Copenhagen, Copenhagen DK-2200, Denmark.; ^2^Biotech Research and Innovation Centre, Faculty of Health and Medical Sciences, University of Copenhagen, Copenhagen DK-2200, Denmark.; ^3^Novo Nordisk Foundation Centre for Stem Cell Biology, DanStem, Faculty of Health and Medical Sciences, University of Copenhagen, Copenhagen DK-2200, Denmark.; ^4^Department of Hematology, Rigshospitalet/National University Hospital, University of Copenhagen, Copenhagen DK-2200, Denmark.; ^#^Authors contributed equally.

**Keywords:** DNA damage response, PI3K/AKT, chemotherapy, resistance, AML

## Abstract

Resistance of cancer patients to DNA damaging radiation therapy and chemotherapy remains a major problem in the clinic. The current review discusses the molecular mechanisms of therapy resistance in acute myeloid leukemia (AML) conferred by cooperative chemotherapy-induced DNA damage response (DDR) and mutational activation of PI3K/AKT signaling. In addition, strategies to overcome resistance are discussed, with particular focus on studies underpinning the vast potential of therapies combining standard chemotherapy AML regimens with small molecule inhibitors targeting key regulatory hubs at the interface of DDR and oncogenic signaling pathways.

## INTRODUCTION

Resistance to conventional chemo- and radiotherapy is a mainstay of many cancer entities and a major obstacle in the clinic. Thus, understanding the molecular mechanisms of resistance in cancer patients is essential to define relevant druggable targets for therapeutic interventions and overcome therapy resistance.

Acute myeloid leukemia (AML) patients exhibit a dismal overall survival (OS) rate (five-year OS 25%) due to primary resistance or relapse when treated with current standard AML chemotherapy regimens^[1,2]^. Survival remains particularly poor for elderly/unfit and relapsed/refractory (R/R) AML patients, who rarely survive beyond two years^[1-3]^.

AML emerges through sequential acquisition of genetic aberrations comprising a few drivers that partially promote abnormal activity of DNA damage response (DDR) and “oncogenic” signaling pathways, which together confer DNA repair, survival, proliferation, and ultimately therapy resistance toward current AML therapies^[2,4-8]^. Thus, inhibition of drug resistance conferred by aberrant mutational activation of DDR and signaling pathways holds great potential to improve therapy response and OS of AML patients.

The current review deals with chemotherapy resistance in AML with particular focus on studies highlighting the potential of targeting DDR and phosphoinositide 3-kinase (PI3K)/protein kinase B (AKT) signaling pathways to overcome resistance in AML patients. For detailed information on therapeutic strategies targeting DDR and PI3K/AKT signaling pathways in other types of cancers, we refer to recent comprehensive reviews^[9-21]^.

## AML THERAPY

Standard curative treatment of younger/fit AML patients (< 65-75 years) consists of intensive induction chemotherapy encompassing an antimetabolite (i.e., cytarabine) and a DNA damaging anthracycline (i.e., daunorubicin, doxorubicin, idarubicin, *etc*.) (in the following, cytarabine/anthracycline regimens are referred to as standard AML chemotherapy)^[1,2,22]^. Following induction therapy, AML patients will proceed with consolidation therapy, comprising additional cycles of intensive chemotherapy or allogeneic stem cell transplantation, dependent on the individual AML patient’s age, fitness, and genetic risk stratification^[1,2]^. Currently, only 40% of younger and fit AML patients are long-term survivors (i.e., five-year OS 40%), as the majority relapse or exhibit primary resistance (R/R) toward current intensive therapeutic regimens^[1,2]^. Of the remaining elderly/unfit (> 75 years) AML patients, who are not eligible for intensive standard AML chemotherapy, the vast majority do not survive two years (< 10%) due to limited clinical response to current non-curative standard regimens. These include best supportive care with or without hydroxurea, low-dose cytarabine (LDAC, CR/CRi 11%-19%, median OS < 6 months), and hypomethylating agents (HMAs, namely azacitidne/decitabine, CR/CRi 27%, median OS 10.5 months), which also cause some degree of DNA damage^[1,2,23]^. More recent studies have highlighted the substantial therapeutic potential of the anti-apoptotic BCL2 inhibitor venetoclax in combination with current standard AML regimens. More specifically, a seminal phase III trial combining azacytidine with venetoclax demonstrated significant improvement of response rates and survival of elderly/unfit AML patients as compared to single treatment with azacytidine alone (AZA/VEN *vs*. single AZA: CR/CRi 66.4% *vs*. 28.3%, median OS 14.7 *vs*. 9.6 months)^[24]^. Consistently, preliminary studies combining the hypomethylating agent decitabine (DEC) or azacytidine with venetoclax for R/R AML patients have demonstrated improved response rates (DEC/VEN or AZA/VEN, CR/CRi/MLFS 64%) and survival rates (DEC/VEN or AZA/VEN, one-year OS 53%, median OS not reached) as compared to single treatment with azacytidine or decitabine (CR/CRi 16%, median OS 6.7 months) and similar response rates when compared to treatment with conventional intensive AML salvage chemotherapy (FLAG-IDA, CR/CRi 52%, median OS 10 months)^[25-27]^.

## RATIONALE FOR THERAPEUTIC TARGETING OF DDR IN AML

**Figure 1:**
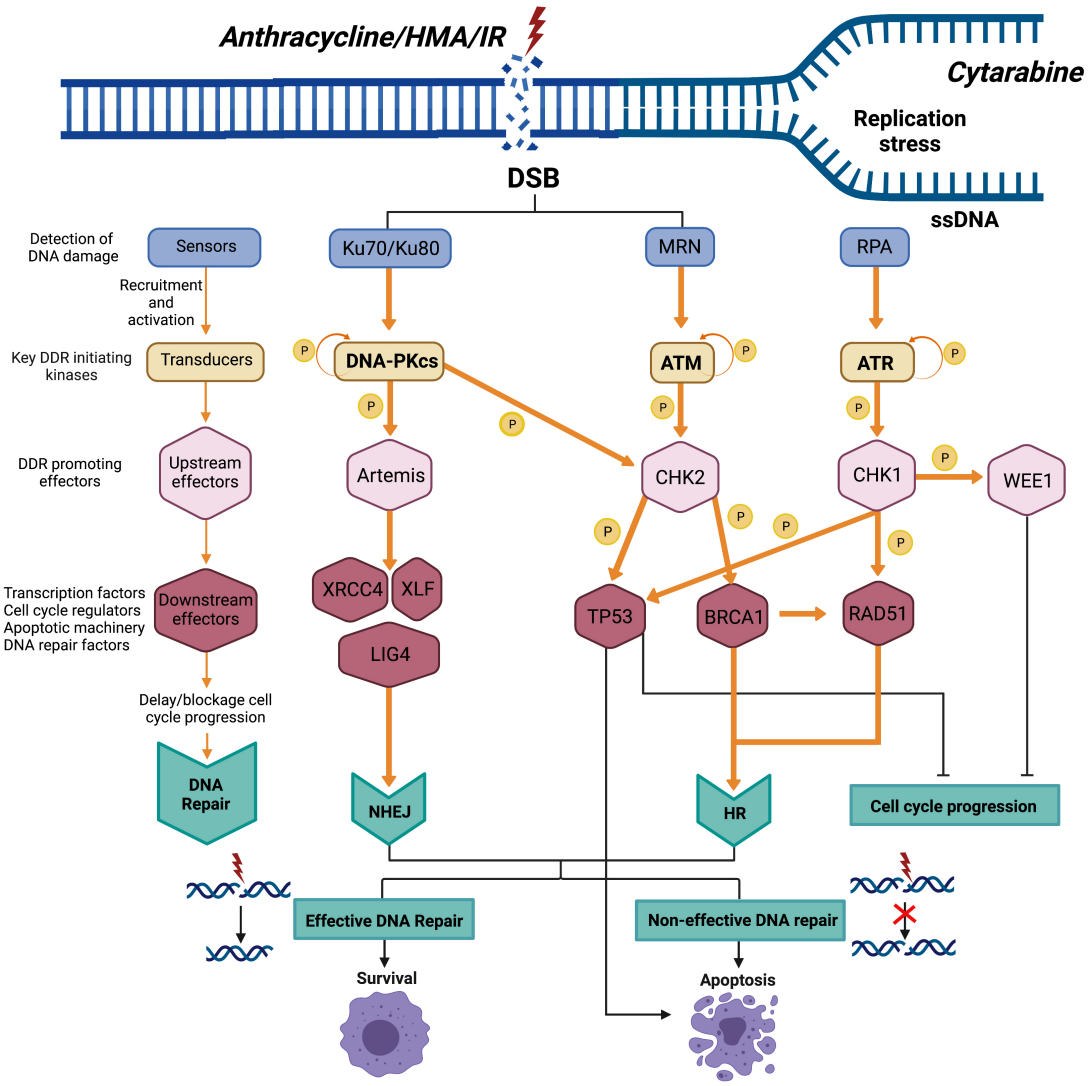
Schematic view of key DNA damage response pathways. Chemo- and radiotherapies (CT and IR) cause DNA damage, which launches a DNA damage response (DDR) to repair DNA and ensure survival of cancer cells. Current AML standard therapies include cytarabine, anthracyclines, or hypomethylating agents (HMAs) such as azacytidine and decitabine. Cytarabine induces stalled replication forks, leading to DDR activation, which promotes survival via the ATR/CHK1 axis and their downstream targets such as WEE1 in AML cells. Anthracyclines and to some extent HMAs such as azacitidine induce DSBs, leading to DDR activation and repair of DSB by HR and NHEJ, respectively. HR and NHEJ are tightly coordinated by the DDR-initiating master regulators ATM and DNA-PK, respectively, which through various DDR downstream substrates promote delay or block of cell cycle progression and repair of DNA, or TP53-mediated apoptosis if DNA is irreversibly damaged. Hence, in respect of outcome, chemotherapy-induced DNA damage and resultant DDR will confer either survival or apoptosis depending on whether the level of cytotoxicity can overcome the capacity of AML cells to repair DNA. IR: Irradiation/radiotherapy; CT: chemotherapy; DSB: double-strand break; P: phosphorylation; HR: homologous recombination; NHEJ: non-homologous end-joining; ATM: ataxia telangiectasia mutated; ATR: ATR serine/threonine kinase; CHK1: checkpoint kinase 1; CHK2: checkpoint kinase 2; DDR: DNA damage response; DNA-PK: DNA-dependent protein kinase catalytic subunit; DSB: double-strand breaks; HMAs: hypomethylating agents; MRN: Mre11, Rad50, and Nbs1 complex; TP53: tumor protein 53; BRCA1: BRCA1 DNA repair associated; WEE1: WEE1 G2 checkpoint kinase; XLF: XRCC4-like factor; XRCC4: X-ray repair cross complementing 4.

Many current cancer therapies cause replicative stress, leading to DNA damage. The latter launches a DDR [Figure 1], which in respect of outcome results in either survival or apoptosis depending on whether the level of cytotoxicity can overcome the capacity of cancer cells to repair DNA^[8,10,11,17,19,28]^. Hence, twisting the balance of therapy-induced DDR from survival toward apoptosis by inhibiting key regulators of the DDR, represents an attractive strategy to enhance sensitivity and overcome therapy resistance in cancer patients^[8,10,11]^. Consistently, recent studies have highlighted the therapeutic potential of DDR inhibitors for the treatment of solid cancers and AML^[29-34]^.

Current AML standard therapies include cytarabine, anthracyclines, and HMAs such as azacytidine and decitabine. Cytarabine induces stalled replication forks leading to DDR activation promoting survival via the DDR master regulator ATR serine/threonine and its downstream substrates such as check point kinase 1 (CHK1) and WEE1 G2 checkpoint kinase (WEE1). Consistently, combinations of cytarabine and ATR inhibitors have demonstrated combinatorial therapeutic effect in some but not all studies on AML^[35,36]^.

Anthracyclines and to some extent HMA induce DNA double-strand breaks (DSB), leading to DDR activation and repair of DSB by homologous recombination (HR) and non-homologous end-joining (NHEJ), respectively^[22,37]^. HR and NHEJ are tightly orchestrated by the DDR master regulators ataxia telangiectasia mutated (ATM) and DNA-dependent protein kinase, catalytic subunit (DNA-PK), respectively, which through various DDR downstream substrates promote: (1) delay or block of cell cycle progression; (2) repair of DNA; or (3) TP53-mediated apoptosis if DNA is irreversibly damaged^[8,11,38]^. Hence, given the high rate of relapse and primary resistance of AML patients, there is a compelling rationale to combine DNA damaging standard AML chemotherapy and HMAs, with inhibitors of the DDR master regulators ATR, ATM, DNA-PK, and their DDR downstream substrates, to boost therapeutic efficacy and overcome therapy resistance.

## RATIONALE FOR THERAPEUTIC TARGETING OF PI3K/AKT SIGNALING

Mutational activation of the PI3K/AKT signaling pathway is a common theme in cancer and is generally associated with a poor response to DNA damaging cancer therapies^[12,13,15,21]^.

Recent studies, mapping the genomic landscape in large AML cohorts, have identified mutations in more than 60% of AML patients, which directly or through intermediaries confer aberrant “oncogenic” activation of the PI3K/AKT signaling pathway [Table 1]. The latter include mutations of receptor tyrosine kinases [KIT proto-oncogene-receptor tyrosine kinase (KIT) and fms related receptor tyrosine kinase 3 (FLT3)], intracellular kinases [e.g., Janus kinase 2 (JAK2)], ASXL transcriptional regulator 1 (ASXL1), and GTPases [e.g., the neuroblastoma RAS viral oncogene homolog (NRAS) Kirsten rat sarcoma viral oncogene homolog (KRAS)], which are all frequently associated with a poor response to standard AML chemotherapy^[5,40-49]^.

**Table 1:** Frequency of mutations associated with PI3K/AKT activation in AML patients

**Mutation**	**Frequency (%)**	**Frequency (%)**
	**(mean age 60 years)^[5]^**	**(mean age 77 years)^[39]^**
***KIT***	6	< 3
***FLT3-ITD/FLT3-TKD***	32	14
***NRAS/KRAS***	23	23
***JAK2***	< 0	5
***ASXL1***	6	10.5
***PTEN***	< 0	< 2

PI3K: Phosphoinositide 3-kinase; AKT: protein kinase B; KIT: KIT proto-oncogene-receptor tyrosine kinase; FLT3: receptor tyrosine kinase 3; NRAS: neuroblastoma RAS viral oncogene homolog; KRAS: Kirsten rat sarcoma viral oncogene homolog; JAK2: Janus kinase 2; ASXL1: ASXL transcriptional regulator 1; PTEN: phosphatase and tensin homolog.

Upon activation, PI3Ks and their second messenger phosphatidylinositol 3-phosphate promote phosphoinositide-dependent protein kinase-1 (PDPK1)- and mTOR complex 2 (mTORC2)-dependent phosphorylation of AKT at its T308 and S473 residues, respectively^[20,50]^. AKT itself activates numerous downstream targets including mTOR complex 1 (mTORC1), glycogen synthase kinase 3 (GSK3), and forkhead box proteins (FOXO). Together, PI3K/AKT downstream signaling directs: (1) proliferation; (2) survival; (3) glucose metabolism; and (4) DNA repair^[13,21,51,52]^. Notably, PI3K/AKT exerts these activities partially through: (1) activation of MDM2-promoted TP53 degradation; (2) regulation of DDR molecule activities (CHK1 and BRCA1); and (3) upregulation of the anti-apoptotic MCL1 and BCL2 proteins through the cyclic adenosine monophosphate response element-binding protein (CREBBP)^[51-56]^. Because PI3K/AKT-dependent MCL1 expression can promote resistance to the recently approved BCL2 inhibitor venetoclax in patients with AML, simultaneous targeting of anti-apoptotic proteins such as MCL1, BCL2, or BCL2L1 (BCL-xL) and PI3K/AKT signaling might cooperatively boost apoptotic activity in AML cells. Indeed, this is corroborated by preclinical PDX trials of solid cancers and AML, highlighting the therapeutic benefit of PI3K/AKT inhibitors in combination with the BCL2 inhibitor venetoclax^[57-59]^. Since the majority of patients with AML will either not respond or in time develop resistance to venetoclax/azacytidine treatment^[24]^, there is a strong reason to explore the therapeutic efficacy of combination therapies including venetoclax and PI3K/AKT inhibitors alone or in combination with standard AML chemotherapy/HMAs.

In conclusion, the frequent mutational activation of PI3K/AKT signaling in AML patients, as well as its prominent role in therapy resistance, provides a rationale for targeted inhibition of PI3K/AKT signaling to enhance the efficacy of standard AML therapies and ultimately overcome therapy resistance.

## CROSSTALK BETWEEN DDR AND PI3K/AKT PATHWAYS

**Figure 2:**
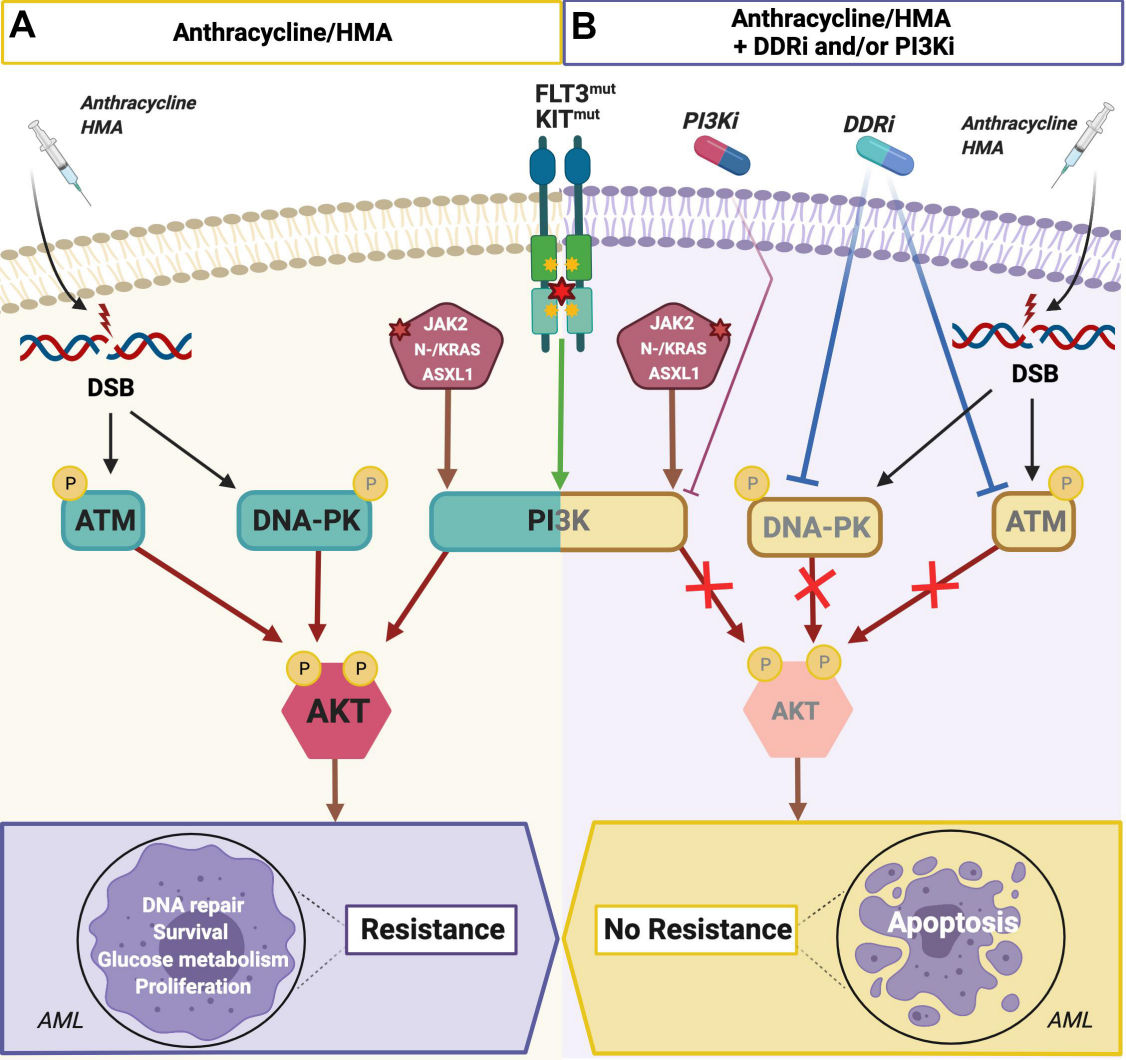
Rationale for combinatorial treatment of AML exhibiting mutational activation of PI3K/AKT signaling with inhibitors of DNA-PK and/or PI3K/AKT and DSB-inducing AML chemotherapeutics (i.e., anthracyclines and HMAs). (A) AML cells harboring mutations in KIT, FLT3, JAK2, ASXL1, or NRAS/KRAS frequently exhibit constitutive “oncogenic” signaling including activation of the PI3K/AKT signaling pathway. Standard therapies for AML patients consist of: (1) cytarabine/anthracycline (dauno-, ida-, or doxorubicin) chemotherapy; or (2) HMAs such as azacytidine, which induce DSBs. Emerging therapy-induced DSBs launch a DDR partly via DNA-PK- and/or ATM-dependent complementary enhancement of AKT downstream signaling, which promotes: (1) proliferation; (2) survival; (3) glucose metabolism; (4) DNA repair; and, ultimately (5) therapy resistance. (B) Simultaneous treatment with inhibitors of DDR or PI3K/AKT signaling in combination with an anthracycline/HMA abrogates AKT downstream signaling and DNA repair, leading to increased DNA damage, apoptosis of AML cells, and ultimately better therapy response and clinical outcome. ATM: Ataxia telangiectasia mutated; ATR: ATR serine/threonine kinase; DDR: DNA damage response; DSBs: double-strand brakes; FLT3: fms related receptor tyrosine kinase 3; HMAs: hypomethylating agents; KIT: KIT proto-oncogene-receptor tyrosine kinase.

There is compelling evidence of crosstalk activation and regulation between DDR and “oncogenic” signaling pathways including the PI3K/AKT signaling pathway [Figure 2]^[60,61]^. Studies have shown that AKT is directly phosphorylated (i.e., activated) by the DDR master regulators DNA-PK and ATM upon therapeutic DNA damage^[21,60,62]^. The latter marks the PI3K/AKT axis as a key regulatory hub, which enhances DNA repair and survival in response to DNA-PK or ATM cross-activation, through collateral regulation of DNA repair and inhibition of TP53-dependent apoptosis^[21,53]^. Consistently, targeted inhibition of PI3K/AKT signaling can confer replicative stress and cell death of cancer cells, underpinning its role in collateral enhancement of the DDR-mediated resistance to DNA damaging therapies^[18,63]^. Overall, these findings highlight the potential of therapies combining DDR and/or PI3K/AKT inhibitors with conventional therapies conferring replicative stress and DNA damage.

## PRECLINICAL STUDIES OF DDR AND PI3K/AKT INHIBITOR COMBINATION THERAPIES

Mutational activation of the PI3K/AKT signaling pathway in AML patients is associated with poor clinical outcome when treated with intensive AML chemotherapy regimens including cytarabine and anthracyclines. The latter is illustrated by a significantly higher relapse rate of t(8;21) and inv(16) AML patients harboring gain-of-function KIT mutations *vs*. wild-type KIT^[64-67]^. In a recent study, Estruch *et al.*^[68]^ applied a genetically modified inv(16)/KIT^D816Y^ AML mouse model, mimicking AML patients with constitutive mutational KIT-dependent activation of the PI3K/AKT signaling pathway, to explore the therapeutic efficacy of standard AML chemotherapy in combination with DDR or PI3K/AKT inhibitors (i.e., DNA-PKi/NU7026 or pan-PI3Ki/BKM120). Treatment of inv(16)/KIT^D816Y^ AML cells with chemotherapy markedly increased activation of AKT via DNA-PK-dependent phosphorylation. Complementary mechanistic experiments further demonstrated that chemotherapy in combination with either PI3K or DNA-PK inhibitors significantly decreased chemotherapy-induced activation of AKT signaling, leading to a significant increase of DNA damage and apoptosis of inv(16)/KIT^D816Y^ AML cells. Subsequent treatment, with a PI3K or a DNA-PK inhibitor in combination with standard AML chemotherapy, synergistically inhibited *in vitro* growth and survival of AML cells in clonogenic assays. Consistently, combination of standard AML chemotherapy and these inhibitors significantly improved overall survival of inv(16)/KIT^D816Y^ AML mice in preclinical trials compared to single treated or untreated mice. Intriguingly, treatment with the PI3K/AKT inhibitor alone abrogated PI3K signaling activity and exhibited some therapeutic activity in clonogenic assays and preclinical trials, whereas single treatment with the DNA-PK inhibitor elicited no therapeutic effect due to lack of DNA-PK activation in absence of chemotherapy-induced DNA damage.

In agreement with these findings, Ueno *et al*.^[69]^ recently demonstrated that simultaneous treatment with a FLT3 inhibitor (i.e., gilteritinib) and standard AML chemotherapy markedly enhanced chemotherapy response in FLT3-ITD mutant AML patient-derived xenografts.

Together, these preclinical studies suggest that DNA repair and survival of AML cells mediated by therapy-induced activation of a DDR and its collateral enhancement of constitutive mutational PI3K/AKT activation represents a key resistance mechanism in AML patients treated with DNA damaging therapies. Both studies further suggest that AML patients, who have a high mutational PI3K/AKT signaling activity potentially will benefit from combinatorial treatment with chemotherapy and DDR inhibitors and/or direct PI3K/AKT inhibitors or, alternatively, inhibitors of upstream PI3K/AKT activators such as KIT, FLT3, JAK2, ASXL1, and NRAS/KRAS. Since both standard AML induction chemotherapy and HMAs induce DSBs, which boost PI3K/AKT signaling in a DDR-dependent manner, the majority of AML patients treated with curative or non-curative therapeutic regimen will potentially benefit from complementary treatment with relevant small molecule inhibitors.

## CLINICAL STUDIES ADVOCATING THE THERAPEUTIC POTENTIAL OF DDR AND PI3K/AKT INHIBITOR COMBINATION THERAPIES

Currently, DDR inhibitors remain to be approved for treatment of AML patients. However, several DDR inhibitors are currently being tested in clinical trials of solid cancers and AML, alone or in combination with DNA damaging and cytotoxic chemotherapy and radiation therapies, including CC-115 (DNA-PKi/MTORi), nedisertib/M3814 (DNA-PKi), VE-821 (ATMi/ATRi), and VX-984 (DNA-PKi)^[10,70]^. Moreover, two recent studies depicted in Table 2 confirm that DDR inhibitors M6620/VX-970 (ATRi) and AZD1775 (Wee1i) enhance the efficacy of chemotherapies in patients with solid cancers^[33,71]^.

**Table 2:** Selected clinical trials combining direct/indirect PI3K inhibitors or DNA damage response inhibitors with chemotherapy or hypomethylating agents

**Compound**	**Direct/indirect PI3Ki**	**DDRi**	**Study design**	**Indication**	**Combination therapy**	**Clinical trial Ref.**	**Clinical outcome**
**Quizartinib**	FLT3 inhibitor	-	Phase I	*de novo *FLT3mut AML	Q + standard CT *vs*. standard CT	NCT 01390337^[70]^	ORR 84% (16/19 patients, 14 patients CRc + 2 patients MLFS). No additional toxicity.
**Midostaurin**	FLT3 inhibitor	-	Phase III	*de novo *FLT3mut AML	M + standard CT *vs*. standard CT	NCT00651261^[68]^	OS 74.7 months (midostaurin) *vs. *25.6 months (placebo), *P *= 0.009), median EFS 8.2 mo (midostaurin) and 3.0 mo (placebo) *P *= 0.002)
**Idelalisib**	PI3K delta inhibitor	-	Phase III	R/R chronic lymphocytic leukaemia	I + bendamustine/rituximab *vs*. bendamustine/rituximab	NCT01569295^[71]^	Median PFS 20.8 months (idelalisib) *vs*. 11.1 months (placebo), *P *< 0.0001
**Tucatinib**	HER2 inhibitor	-	Phase II	HER2-positive metastatic breast cancer	T + trastuzumab/capecitabine *vs*. trastuzumab/capecitabine	NCT02614794^[72]^	2-year OS at 2 44.9% (tucatinib) *vs*. 26.6% (placebo), *P *< 0.005
**Berzosertib (VX-970, M6620)**	-	ATR inhibitor	Phase II	Platinum-resistant high-grade serous ovarian cancer	B + gemcitabine *vs*. gemcitabine	NCT02595892^[66]^	Median PFS 22.9 weeks (berzosertib) *vs*. 14.7 weeks (placebo), *P *= 0.044
**AZD1775**	-	Wee1 inhibitor	Phase II	TP53mut ovarian cancer, R/R to first-line platinum-based therapy	A + carboplatin	NCT01164995^[65]^	Median PFS 5.3 months, OS 12.6 months. Clinical proof that Wee1 inhibitor enhances carboplatin efficacy in TP53-mutated tumors

ATR: ATR serine/threonine kinase; FLT3: rms telated teceptor tyrosine Kinase 3; HER2: human epidermal growth factor receptor 2; PI3K: phosphoinositide 3-kinase; R/R: relapsed/refractory; TP53: tumor protein P53; WEE1: WEE1 G2 checkpoint kinase; CRc: composite complete remission; EFS: event-free survival; ORR: overall response rate; MLFS: morphological leukemia-free state; OS: overall survival; PFS: progression-free survival.

Even though several Phase I/II clinical trials have tested combinations of direct PI3K/AKT inhibitors and chemo- or radiotherapies in patients with solid cancers and AML, no inhibitors have thus far been approved for single or combinatorial treatment of AML patients^[12,72]^. However, recent studies have demonstrated that simultaneous treatment with FLT3 inhibitors, which potentially block mutational PI3K/AKT downstream signaling, significantly improved therapeutic efficacy of: (1) HMAs in elderly/unfit FLT3 mutant AML patients (azacytidine/gilteritinib); and (2) standard AML chemotherapy in younger FLT3 mutant AML patients (cytarabine/anthracycline/midostaurin or cytarabine/anthracycline/quizartinib) [Table 2]^[73-75]^. This potential therapeutic effect of combinatorial treatment is further supported by a seminal clinical trial demonstrating that simultaneous treatment of patients with R/R chronic lymphatic leukemia with the PI3K inhibitor idelalisib and alkylating agent/antibody (i.e., bendamustine/rituximab), significantly improved the median progression-free survival (PFS) as compared to treatment with alkylating agent/antibody alone (median PFS 20.8 months *vs*. 11.1 months, *P *< 0.0001) [Table 2]^[76]^. Consistently, women with HER2-positive metastatic breast cancer markedly improved survival in response to triple treatment with a HER2 small molecule inhibitor (tucatinib), an anti-HER-antibody inhibiting ligand binding to HER (trastuzumab), and an antimetabolite (capecitabine) as compared to dual treatment with antibody/antimetabolite (two-year OS 44.9% *vs*. 26.6%, *P *= 0.005) [Table 2]^[77]^.

In conclusion, clinical trials highlight the therapeutic potential of DDR and PI3K/AKT inhibitor combination therapies and advocate for future AML trials investigating the efficacy of dual or triple combination therapies including DDR and/or PI3K inhibitors in combination with either chemotherapy or HMAs.

## PERSPECTIVES AND CHALLENGES OF DDR AND PI3K/AKT INHIBITOR COMBINATION THERAPIES

Conceptually, cancer poly-therapies should combine drugs which: (1) exhibit additive or synergistic therapeutic activity; (2) target distinct cancer vulnerabilities to circumvent the development of resistance; and (3) can be administered at active therapeutic doses without surpassing clinically manageable levels of toxicity. Hence, the hallmark of cancer therapies combining novel drugs with standard therapies relates to whether their higher therapeutic efficacy outweighs the increase of toxicity and ultimately leads to a significant clinical benefit. The latter is particularly relevant in AML, considering the substantial toxicity of standard AML induction chemotherapies in younger AML patients as well as HMAs in elderly/unfit AML patients. Thus, the balance of benefit and toxicity is key to the design of clinical trials combining DDR and/or PI3K/AKT inhibitors with current AML therapies in terms of drug doses, duration of drug administration during treatment cycles, concomitant *vs*. sequential drug administration, and finally the length of treatment cycles. In this respect, recent experimental and clinical studies underscore that inhibitors of DDR and PI3K/AKT signaling pathways including FLT3 inhibitors should be administered simultaneously rather than sequentially with chemotherapy/HMAs to potentiate rather than complement therapeutic efficacy, as is the case with AML treatment regimens and ongoing trials combining standard AML chemotherapy with sequential inhibitor administration^[73,75]^. Although simultaneous treatment of AML patients with chemotherapy/HMAs and complementary drugs frequently comes with enhanced toxicity, primarily in the form of prolonged cytopenia and associated neutropenic infections, recent AML studies have demonstrated manageable toxicity and overall clinical benefit of standard AML therapies in combination with simultaneous administration of novel drugs^[24,78-80]^. However, future clinical trials are needed to define the optimal drug dosages for combination therapies encompassing a backbone of standard AML chemotherapy or HMAs and inhibitors of DDR and/or PI3K/AKT signaling pathways. Ideally, such clinical trials should be accompanied by exploratory biomarker analyses for guided treatment of responders and prevention of overtreatment of non-responders. Such biomarker assessment should include NGS-based genomics and complementary functional analyses such as drug screening and phosphoproteomics to predict AML patients responding to specific inhibitors of DDR and PI3K/AKT signaling pathways alone or in combination with chemotherapy/HMAs.

## CONCLUSION

Oncogenic DDR and PI3K/AKT signaling activity is a common theme in various cancers, including AML, and is frequently associated with therapy resistance and poor clinical outcome. Hence, therapeutic targeting of DDR and PI3K/AKT signaling pathways alone or in combination with current standard AML chemotherapy and HMAs stands out as a potential strategy to overcome resistance and improve clinical outcome of AML patients. In a broader perspective, current findings highlight the vast potential of combining conventional DNA-damaging therapies with inhibition of signaling molecules at the interface of DDR and oncogenic signaling pathways to overcome therapy resistance and improve clinical outcome in patients with AML as well as other cancer entities.

## DECLARATIONS

### Availability of data and materials

All data and materials included in this review are available online.

### Copyright

© The Author(s) 2021.
